# Continued high incidence of children with severe influenza A(H1N1)pdm09 admitted to paediatric intensive care units in Germany during the first three post-pandemic influenza seasons, 2010/11–2012/13

**DOI:** 10.1186/s12879-015-1293-1

**Published:** 2015-12-18

**Authors:** Andrea Streng, Christiane Prifert, Benedikt Weissbrich, Johannes G. Liese, for the Bavarian PICU Study Group on Influenza and Other Viral ARI

**Affiliations:** Department of Paediatrics, University of Würzburg, Josef-Schneider-Str. 2, 97080 Würzburg, Germany; Institute of Virology and Immunobiology, University of Würzburg, Würzburg, Germany

**Keywords:** Influenza, Children, Intensive care, Post-pandemic

## Abstract

**Background:**

Previous influenza surveillance at paediatric intensive care units (PICUs) in Germany indicated increased incidence of PICU admissions for the pandemic influenza subtype A(H1N1)pdm09. We investigated incidence and clinical characteristics of influenza in children admitted to PICUs during the first three post-pandemic influenza seasons, using active screening.

**Methods:**

We conducted a prospective surveillance study in 24 PICUs in Bavaria (Germany) from October 2010 to September 2013. Influenza cases among children between 1 month and 16 years of age admitted to these PICUs with acute respiratory infection were confirmed by PCR analysis of respiratory secretions.

**Results:**

A total of 24/7/20 influenza-associated PICU admissions were recorded in the post-pandemic seasons 1/2/3; incidence estimates per 100,000 children were 1.72/0.76/1.80, respectively. Of all 51 patients, 80 % had influenza A, including 65 % with A(H1N1)pdm09. Influenza A(H1N1)pdm09 was almost absent in season 2 (incidence 0.11), but dominated PICU admissions in seasons 1 (incidence 1.35) and 3 (incidence 1.17). Clinical data was available for 47 influenza patients; median age was 4.8 years (IQR 1.6–11.0). The most frequent diagnoses were influenza-associated pneumonia (62 %), bronchitis/bronchiolitis (32 %), secondary bacterial pneumonia (26 %), and ARDS (21 %). Thirty-six patients (77 %) had underlying medical conditions. Median duration of PICU stay was 3 days (IQR 1–11). Forty-seven per cent of patients received mechanical ventilation, and one patient (2 %) extracorporeal membrane oxygenation; 19 % were treated with oseltamivir. Five children (11 %) had pulmonary sequelae. Five children (11 %) died; all had underlying chronic conditions and were infected with A(H1N1)pdm09. In season 3, patients with A(H1N1)pdm09 were younger than in season 1 (*p* = 0.020), were diagnosed more often with bronchitis/bronchiolitis (*p* = 0.004), and were admitted to a PICU later after the onset of influenza symptoms (*p* = 0.041).

**Conclusions:**

Active screening showed a continued high incidence of A(H1N1)pdm09-associated PICU admissions in the post-pandemic seasons 1 and 3, and indicated possible underestimation of incidence in previous German studies. The age shift of severe A(H1N1)pdm09 towards younger children may be explained by increasing immunity in the older paediatric population. The high proportion of patients with underlying chronic conditions indicates the importance of consistent implementation of the current influenza vaccination recommendations for risk groups in Germany.

## Background

Influenza is one of the most common vaccine-preventable viral diseases, with the highest morbidity reported for children and elderly patients [1, 2]. Influenza infections during childhood usually present as mild respiratory upper airway disease, but severe complications and fatalities also occur, especially in children less than 2 years of age and in children with underlying chronic conditions [2–7]. However, 40–50 % of influenza-associated fatalities occur in previously healthy children [4, 8].

Before the influenza A(H1N1)pdm09 pandemic in 2009/2010, comparisons of clinical characteristics between patients infected with different influenza types (A vs. B) and between patients infected with different influenza A subtypes showed only small differences when controlling for age [9, 10]. During the pandemic, however, some studies observed increased morbidity and mortality among children compared to previous seasonal influenza [6, 11–13], while other studies described the clinical features of A(H1N1)pdm09 as being similar or even milder [14, 15]. Acute respiratory distress syndrome (ARDS) and fatal viral pneumonia was observed more frequently during the pandemic [16]. Post-pandemic surveillance was recommended, as circulation of A(H1N1)pdm09 was expected to continue for several years, gradually assuming a seasonal influenza pattern [16].

In Germany, influenza sentinel surveillance on outpatients of all ages [17] confirmed that the first post-pandemic season 2010/11 was dominated by influenza A(H1N1)pdm09 (62 %), co-circulating with influenza B (37 %) whereas A(H3N2) was rare (<1 %). During the second season 2011/12, A(H1N1)pdm09 was rare (1 %), whereas A(H3N2) was diagnosed in 75 % of cases and co-circulated with influenza B (24 %). In the third season 2012/13, all three types/subtypes co-circulated in similar proportions (34 % A(H1N1)pdm2009, 31 % A(H3N2), and 35 % B).

Information on the incidence and clinical characteristics of severe paediatric influenza resulting in intensive care treatment and/or fatal outcome is still limited in Germany, and post-pandemic data is thus far available only for the season 2010/11 [18–20]. Based on cases recorded by a nation-wide paediatric intensive care unit (PICU) reporting system, the pre-pandemic (2005/06–2007/08), pandemic (2009/10) and post-pandemic (2010/11) annual incidence of severe influenza cases per 100,000 children below 15 or 17 years of age was estimated as 0.05, 0.8–1.0, and 0.4, respectively [18–20]. The data so far available indicated a shift towards younger children in A(H1N1)pdm09 cases from the pandemic to the first post-pandemic season [20]. In these studies, it remained unclear whether the higher pandemic and post-pandemic incidence in children was caused by higher influenza activity, heightened physician awareness, more frequent or more sensitive influenza testing, or a more severe course of disease of A(H1N1)pdm09 [18]. Furthermore, all these previous studies may have been affected by under-reporting, as influenza cases were reported at the discretion of the PICU physician without systematic screening for influenza in patients with severe acute respiratory infection.

In the study presented here, we used active screening to estimate the incidence of laboratory-confirmed influenza-associated PICU admissions in one of Germany’s largest federal states during the first three post-pandemic seasons. Furthermore, we described the clinical characteristics of influenza PICU patients and compared patients with severe A(H1N1)pdm09 disease between the post-pandemic seasons.

## Methods

### Study population and setting

Prospective, active surveillance was conducted in PICUs of paediatric hospitals in Bavaria, Germany. On December 31^st^, 2010 roughly 2,001,700 children <17 years of age were registered in Bavaria [21], representing 16 % of the German population in this age group [22]. The annual study population was defined as the sub-group of all children in Bavaria at least 1 month and <17 years of age.

All 30 paediatric hospitals in Bavaria equipped for paediatric intensive care treatment of children older than 1 month of age were invited to participate. These PICUs reported a total of 432 intensive care beds (median 14, IQR 11–16), including 207 beds (median 9, IQR 6–12) equipped with ventilation facilities.

### Case definition and surveillance system

From October 2010 to September 2013 (season 1: Oct 2010–Sep 2011, season 2: Oct 2011–Sep 2012, season 3: Oct 2012–Sep 2013), all patients who fulfilled the following inclusion criteria were enrolled: *i)* admission to a participating PICU with suspected acute respiratory infection (ARI) of the upper or lower respiratory tract, with ARI-related symptoms (for example, coryza, cough, or sore throat); *ii)* age at PICU admission due to ARI at least 1 month and below 17 years of age; *iii)* parental written informed consent. Enrolled children with PCR-confirmed influenza were classed as influenza-associated ARI.

The PICU physician documented demographic characteristics, underlying chronic medical conditions, influenza vaccination status, diagnostic findings, ARI-associated diagnoses and complications, treatment, duration of hospital and PICU stay, and outcome in a structured questionnaire. A respiratory sample, usually a flocked nasopharyngeal or pharyngeal swab, was collected on the day of PICU admission for PCR-confirmation of influenza. Microbiological testing for bacteria or fungi was at the discretion of the PICU physician; pathogens detected at usually sterile sites or in tracheal aspirates were classified as bacterial or fungal co-infection.

### PCR confirmation of influenza

PCR confirmation of influenza was performed either at the local laboratories of the participating PICUs using influenza-specific PCR, or (in the majority of cases) at the central laboratory at the Institute of Virology and Immunobiology of the University of Würzburg using multiplex PCR for respiratory viruses. For the latter, respiratory samples were placed in a viral transport medium (Mast Diagnostica GmbH, Reinfeld, Germany). At the central laboratory, they were tested using the commercial multiplex PCR ‘FTD® Respiratory pathogens 21’ (Fast Track Diagnostics, Luxembourg) to screen for respiratory viruses (sensitivity and specificity of 99–100 % compared to singleplex PCR assays for all included viruses in clinical samples). Pathogens detected by the test kit included influenza A and B viruses, respiratory syncytial virus (RSV), parainfluenza virus (PIV) 1–4, coronavirus (CoV) NL63, OC43, HKU1, and 229E, human metapneumovirus (hMPV), human bocavirus (hBoV), adenovirus (AdV), rhinovirus (RhV), enterovirus (EV), parechovirus (PV), and additionally *Mykoplasma pneumoniae*. Samples positive for influenza A and B virus RNA in the multiplex PCR were further analysed to determine the subtype and lineage, respectively. Primers and probes specific for influenza A(H1N1)pdm09 were included in the ‘FTD Respiratory pathogens 21’ kit. All samples positive for influenza A virus RNA but negative for influenza A virus (H1N1)pdm09 RNA were tested by a PCR specific for influenza A virus H3.

### Statistical analysis

All data was entered into a Microsoft Access database and transferred to IBM SPSS 21.0 for statistical analysis. Data was analysed descriptively (percentages, or median with inter-quartile range, IQR). Comparisons between groups were assessed for significance (*p* < 0.05, two-sided) using Pearson’s Chi^2^-test or Fisher’s Exact test for categorical data, and the Mann–Whitney *U*-test for continuous data.

The minimum incidence of influenza-associated PICU admissions per 100,000 children <17 years of age was calculated for each season based on the observed number of influenza PICU patients with a residential address in Bavaria. To correct for non-participating PICUs, the estimates of the total number of PICU influenza cases treated in all eligible PICUs in Bavaria per season were derived taking into account the annual percentage of participating PICUs. The annual study population was used as denominator.

A similar questionnaire and case definition had been used in previous studies on influenza-related PICU admission [18, 19]. Key variables were extracted from these publications for comparison purposes. Data from Streng et al. [18] and the present study were pooled for statistical comparison of pre- and post-pandemic seasons.

### Ethical considerations

The study was approved by the Ethical Committee of the Medical Faculty, University of Würzburg, Germany.

## Results

### Participating PICUs and recruitment of ARI patients

During the 3-year-observation period, a total of 24 PICUs participated in the study (80 % of the 30 eligible PICUs in Bavaria), with 20/14/17 PICUs participating in seasons 1/2/3 (67 %/47 %/57 % of all eligible PICUs). Twelve of the 24 hospitals participated in all three seasons. Reasons stated for non-participation in all or single seasons were time limitations or staff shortages at the PICUs. Participating and non-participating PICUs did not differ significantly in the number of intensive care beds. The 24 participating PICUs enrolled a total of 572 (233/151/188) ARI patients (median 13, IQR 5–30).

### Frequency of PICU patients with PCR-confirmed influenza, by type and subtype

Among the 572 enrolled PICU ARI patients, a total of 51 PCR-confirmed influenza patients (24/7/20 in seasons 1/2/3) were reported (Table 1). Influenza cases occurred between December 2010 and March 2011, between February and March 2012, and between December 2012 and April 2013, with 90.2 % of all influenza patients admitted during the months January to March.

**Table 1:** Frequency of PCR-confirmed influenza patients in Bavarian PICUs, 2010–2013. Data are shown by virus type/subtype (*n* = 51)

	Influenza type/subtype					
Seasons	All patients	A	*A(H1N1)pdm09*	*A(H3N2)*	*A (subtype n.a.)*	B^a^
	N (%)	N (%)	*N (%)*	*N (%)*	*N (%)*	N (%)
Season 1 (Oct 2010–Sep 2011)	24 (100.0)	23 (95.8)	*19 (79.2)*	*0 (0.0)*	*4 (16.7)* ^b^	1 (4.2)
Season 2 (Oct 2011–Sep 2012)	7 (100.0)	2 (28.6)	*1 (14.3)*	*1 (14.3)*	*0 (0.0)*	5 (71.4)
Season 3 (Oct 2012–Sep 2013)	20 (100.0)	16 (80.0)	*13 (65.0)*	*3 (15.0)*	*0 (0.0)*	4 (20.0)
Season 1–3 (Oct 2010–Sep 2013)	51 (100.0)	41 (80.4)	*33 (64.7)*	*4 (7.8)*	*4 (7.8)*	10 (19.6)

^a^Influenza B lineages in Season 1/2/3: B-Victoria 0/3/0, B-Yamagata 0/1/1, B without lineage 1/1/3

^b^One patient was diagnosed with both seasonal influenza A (without subtype) and A(H1N1)pdm09 by local PCR, and allocated for further analysis to the group influenza A (subtype n.a.)

Of the 51 influenza patients, 41 (80.4 %) were confirmed by multiplex PCR, and 10 (19.6 %) by local PCR. Forty-one patients (80.4 %) were diagnosed with influenza A, and 10 (19.6 %) with influenza B (Table 1). Influenza A was subtyped as A(H1N1)pdm09 in 33 and as A(H3N2) in four patients; for four further patients, the A subtype was not available or indeterminate. The frequency of influenza types/subtypes differed strongly between observation years (Table 1). Type A was diagnosed in 96 %/29 %/80 % of PICU patients with PCR-confirmed influenza in seasons 1/2/3, respectively, including 79 %/14 %/65 % patients with A(H1N1)pdm09. Influenza B was the dominating type in season 2 (71.4 %).

### Incidence estimates for influenza-associated PICU admittance

Based on the observed cases, the minimum incidence for PCR-confirmed influenza-associated PICU admission per 100,000 children <17 years of age in Bavaria was calculated as 1.15/0.36/1.03 for seasons 1/2/3, respectively. Taking into account that the observed cases were based on data from 67 %/47 %/57 % of all eligible PICUs, the total number of influenza-associated PICU admissions in Bavaria was estimated and corrected incidences were calculated as 1.72/0.76/1.80 per 100,000 children <17 years. Subtype-specific corrected incidences were 1.35/0.11/1.17 for A(H1N1)pdm09, 0.0/0.11/0.27 for A(H3N2), and 0.07/0.54/0.36 for B.

### Clinical characteristics of influenza-associated PICU admissions

#### Demographic characteristics, hospital admittance and length of stay

For 47 (92.2 %) of the 51 PCR-confirmed influenza PICU patients, questionnaires with clinical data were available (23/6/18 in seasons 1/2/3, respectively). Eighty-five per cent of these were diagnosed with influenza A, including 68 % with A(H1N1)pdm09, and 15 % with influenza B. Sixty per cent were male; median age was 4.8 years, with 51.1 % of children <5 years and 23.4 % <1 year of age (Table 2).

**Table 2:** Demographic characteristics and details on PICU/hospital admission of PCR-confirmed influenza patients in Bavarian PICUs, 2010–2013. Data are shown by virus type/subtype (*n* = 47)^a^

	Influenza type/subtype						Comparison of seasons
							A(H1N1)pdm09^c^
Characteristics	All patients	A	*A(H1N1)pdm09*	*A(H3N2)*	*A (subtype n.a.)*	B	S1 vs S3
	N = 47	N = 40	*N = 32*	*N = 4*	*N = 4*	N = 7	*p*-value*
Male sex; n (%)	28 (59.6)	24 (60.0)	*18 (56.3)*	*3 (75.0)*	*3 (75.0)*	4 (57.1)	0.481
Age at ICU admission, in years (median, IQR)	4.8 (1.6–11.0)	4.7 (1.1–11.1)	*5.4 (1.6–11.3)*	*0.9 (0.6–4.4)*	*4.9 (2.3–10.4)*	5.0 (2.3–9.4)	0.020
Interval between onset of ARI symptoms and ICU admission, in days (median, IQR)^b^	3.0 (2.0–5.3)	3.0 (1.0–6.0)	*3.5 (1.0–5.8)*	*2.5 (2.0–10.5)*	*3.0 (n.a.)*	3.0 (1.5–5.0)	0.041
Interval between onset of ARI symptoms and hospital admission, in days (median, IQR)^b^	3.0 (1.0–5.0)	3.0 (1.0–5.0)	*3.0 (1.0–5.0)*	*2.5 (2.0–4.5)*	*2.0 (n.a.)*	3.0 (1.5–4.0)	0.217
Duration of ICU stay, in days (median, IQR)	3.0 (1.0–11.0)	3.5 (2.0–12.5)	*3.0 (1.3–12.3)*	*9.5 (7.3–29.0)*	*6.5 (2.0–16.3)*	1.0 (1.0–11.0)	0.837
Duration of hospital stay, in days (median, IQR)	7.5 (4.0–16.3)	8.0 (4.0–16.0)	*7.0 (4.0–16.0)*	*11.0 (8.8–59.0)*	*13.0 (7.3–20.3)*	5.0 (3.0–22.0)	0.953

*n.a.* not available, *vs* versus

*Fisher’s Exact Test or Mann–Whitney *U*-test, respectively

^a^Clinical data was not available for one influenza A(H1N1)pdm09 and three influenza B patients

^b^Date of ARI symptom onset missing for nine patients

^c^Comparison restricted to influenza A(H1N1)pdm09 patients from Season 1 (S1; Oct10–Sep 11; *n* = 18) and Season 3 (S3; Oct12–Sep 13; *n* = 13)

After onset of ARI symptoms, children were admitted to hospital after a median interval of 3.0 days; 83 % were transferred to the PICU on the day of hospital admission or the following day (Table 2). Two long-term hospitalized children (4.2 %) required PICU treatment due to ARI and were diagnosed with suspected nosocomial influenza A(H1N1)pdm09 infection. Median length of PICU stay was 3.0 days and median length of total hospital stay was 7.5 days (Table 2).

#### Underlying chronic conditions

Underlying chronic medical conditions were reported for a total of 36 influenza PICU patients (76.6 %) (Table 3). Chronic neurological diseases were most frequent (34.0 %), followed by chronic lung disease (25.5 %), preterm birth (21.3 %), cardiac malformations (17.0 %), obesity (10.6 %), genetic disorders (8.5 %), and immunocompromising conditions (8.5 %).

**Table 3:** Underlying chronic medical conditions of PCR-confirmed influenza patients in Bavarian PICUs, 2010–2013. Data are shown by virus type/subtype (*n* = 47)^a^

	Influenza type/subtype						Comparison of seasons
							A(H1N1)pdm09^c^
Underlying medical conditions	All patients	A	*A(H1N1)pdm09*	*A(H3N2)*	*A (subtype n.a.)*	B	S1 vs S3
	N = 47	N = 40	*N = 32*	*N = 4*	*N = 4*	N = 7	*p*-value*
At least one medical condition; n (%)	36 (76.6)	32 (80.0)	*24 (75.0)*	*4 (100.0)*	*4 (100.0)*	4 (57.1)	0.667
Selected conditions							
- Neurological disease; n (%)	16 (34.0)	14 (35.0)	*13 (40.6)*	*1 (25.0)*	*0 (0.0)*	2 (28.6)	1.000
- Chronic lung disease^b^; n (%)	12 (25.5)	12 (30.0)	*11 (34.4)*	*0 (0.0)*	*1 (25.0)*	0 (0.0)	1.000
- Pre-term birth; n (%)	10 (21.3)	9 (22.5)	*8 (25.0)*	*1 (25.0)*	*0 (0.0)*	1 (14.3)	0.210
- Cardiac malformation; n (%)	8 (17.0)	8 (20.0)	*3 (9.4)*	*3 (75.0)*	*2 (50.0)*	0 (0.0)	0.558
- Obesity; n (%)	5 (10.6)	5 (12.5)	*3 (9.4)*	*0 (0.0)*	*2 (50.0)*	0 (0.0)	0.245
- Genetic disorder; n (%)	4 (8.5)	3 (7.5)	*3 (9.4)*	*0 (0.0)*	*0 (0.0)*	1 (14.3)	0.558
- Immunocompromised; n (%)	4 (8.5)	2 (5.0)	*0 (0.0)*	*1 (25.0)*	*1 (25.0)*	2 (28.6)	n.a.
- Renal disease; n (%)	1 (2.1)	1 (2.5)	*1 (3.1)*	*0 (0.0)*	*0 (0.0)*	0 (0.0)	0.419
- Other underlying conditions; n (%)	9 (19.1)	8 (20.0)	*5 (15.6)*	*2 (50.0)*	*1 (25.0)*	1 (14.3)	0.134

*n.a.* not available, *vs* versus

*Fisher’s Exact test or Mann–Whitney *U*-test, respectively

^a^Clinical data was not available for one influenza A(H1N1)pdm09 and three influenza B patients

^b^Including asthma, broncho-pulmonary dysplasia, other chronic lung disease

^c^Comparison restricted to influenza A(H1N1)pdm09 patients from Season 1 (S1; Oct10–Sep 11; *n* = 18) and Season 3 (S3; Oct12–Sep 13; *n* = 13)

#### Influenza vaccination status

Of 36 influenza PICU patients with underlying chronic conditions, four (11.1 %) were too young (<6 months of age) to have been immunized against influenza, and for two patients (5.6 %), data on their influenza vaccination status was unavailable. Twenty-nine (80.6 %) patients from this risk group had not been vaccinated against influenza although they would have been eligible. One immunocompromised child (2.8 %) had been vaccinated in October 2012, but was diagnosed with A(H3N2) in January 2013.

#### Clinical diagnoses

One or more specific influenza-associated diagnoses/complications were reported for 42 (89.4 %) of the 47 children (Table 4). The most frequent diagnosis was influenza-associated pneumonia (61.7 %), followed by bronchitis/bronchiolitis (31.9 %), and secondary bacterial pneumonia (25.5 %). ARDS was reported for 10 (21.3 %) and sepsis for six children (12.8 %); other complications were rare. Thirty-nine of the 47 patients (83.0 %) underwent a chest radiograph.

**Table 4:** Diagnoses/complications of PCR-confirmed influenza patients in Bavarian PICUs, 2010–2013. Data are shown by virus type/subtype (*n* = 47)^a^

	Influenza type/subtype						Comparison of seasons
							A(H1N1)pdm09^b^
Complications	All patients	A	*A(H1N1)pdm09*	*A(H3N2)*	*A (subtype n.a.)*	B	S1 vs S3
	N = 47	N = 40	*N = 32*	*N = 4*	*N = 4*	N = 7	*p*-value*
At least 1 specific complication; n (%)	42 (89.4)	36 (90.0)	*29 (90.6)*	*3 (75.0)*	*4 (100.0)*	6 (85.7)	0.497
Influenza-associated pneumonia; n (%)	29 (61.7)	26 (65.0)	*22 (68.8)*	*1 (25.0)*	*3 (75.0)*	3 (42.9)	1.000
Bronchitis/bronchiolitis; n (%)	15 (31.9)	13 (32.5)	*8 (25.0)*	*3 (75.0)*	*2 (50.0)*	2 (28.6)	0.004
Secondary bacterial pneumonia; n (%)	12 (25.5)	10 (25.0)	*10 (31.3)*	*0 (0.0)*	*0 (0.0)*	2 (28.6)	0.452
ARDS; n (%)	10 (21.3)	9 (22.5)	*7 (21.9)*	*2 (50.0)*	*0 (0.0)*	1 (14.3)	0.099
Sepsis; n (%)	6 (12.8)	6 (15.0)	*4 (12.5)*	*1 (25.0)*	*1 (25.0)*	0 (0.0)	1.000
Febrile convulsions; n (%)	3 (6.4)	2 (5.0)	*2 (6.3)*	*0 (0.0)*	*0 (0.0)*	1 (14.3)	1.000
Encephalitis/encephalopathy; n (%)	1 (2.1)	1 (2.5)	*1 (3.1)*	*0 (0.0)*	*0 (0.0)*	0 (0.0)	1.000
Laryngitis/pseudocroup; n (%)	1 (2.1)	1 (2.5)	*1 (3.1)*	*0 (0.0)*	*0 (0.0)*	0 (0.0)	1.000
Other^c^; n (%)	6 (12.8)	5 (12.5)	*5 (15.6)*	*0 (0.0)*	*0 (0.0)*	1 (14.3)	1.000

*n.a.* not available, *vs* versus

*Fisher’s Exact test

^a^Clinical data was not available for one influenza A(H1N1)pdm09 and three influenza B patients

^b^Comparison restricted to influenza A(H1N1)pdm09 patients from Season 1 (S1; Oct10–Sep 11; *n* = 18) and Season 3 (S3; Oct12–Sep 13; *n* = 13)

^c^Other complications, excluding the pre-defined complications otitis media, status asthmaticus, and myocarditis (for these pre-defined complications, no case was reported)

#### Co-infections

In addition to influenza, laboratory-confirmed co-infections were reported for 16 children (34.0 % out of 47). Of these, seven (14.9 %) had viral, five (10.6 %) bacterial, one (2.1 %) viral/bacterial, two (4.3 %) viral/fungal, and one (2.1 %) bacterial/fungal co-infections. The most frequent co-infecting virus was RSV (seven children). Microbiological testing was documented for 33 patients (70.2 %), with pathogens detected in usually sterile material from two patients (*Escherichia coli* (1)*, Candida* sp. (1)) and from tracheal aspirates of seven intubated patients (*Candida* sp. (2), *Pseudomonas aeruginosa* (2), *Streptococcus* sp. (2), *Enterococcus faecium* (1), *Escherichia coli* (1) and *Moraxella catarrhalis* (1)).

#### Patient treatment and outcome

The majority of the 47 PICU patients were treated intravenously with antibiotics (72.3 %), and with antipyretics (70.2 %) (Table 5). Thirteen children (27.7 %) received antiviral treatment, including nine (19.1 %) patients treated with oseltamivir (four patients received acyclovir, and one patient ganciclovir and valgancyclovir due to other reasons). The median delay between onset of ARI symptoms and oseltamivir treatment was 3.5 days (range 0 to 10 days), with three children receiving treatment within 2 days after symptom onset. Oseltamivir treatment was associated with the presence of a viral or other co-infection (*p* = 0.045), and with a longer stay at the PICU (median 11 days, IQR 2.5–27.5; *p* = 0.043). Thirteen (27.7 %) children each were treated with catecholamines and with inhalation therapy, mainly with bronchodilators. A total of 22 children (46.8 %) required mechanical ventilation; of these, 11 (23.4 %) received only intratracheal ventilation, seven (14.9 %) only CPAP, and four (8.5 %) both. One child (2.1 %) with an immunosuppressed condition, infected with influenza A(H3N2), RSV, Epstein-Barr virus and *Candida* sp., was treated with extracorporeal membrane oxygenation (ECMO).

**Table 5:** Treatment and outcome of PCR-confirmed influenza patients in Bavarian PICUs, 2010–2013. Data are shown by virus type/subtype (*n* = 47)^a^

	Influenza type/subtype						Comparison of seasons
							A(H1N1)pdm09^c^
Treatment	All	A	*A(H1N1)pdm09*	*A(H3N2)*	*A (subtype n.a.)*	B	S1 vs S3
	N = 47	N = 40	*N = 32*	*N = 4*	*N = 4*	N = 7	*p*-value*
Antiviral drugs^b^; n (%)	13 (27.7)	12 (30.0)	*9 (28.1)*	*1 (25.0)*	*2 (50.0)*	1 (14.3)	1.000
Antibiotics i.v.; n (%)	34 (72.3)	31 (77.5)	*25 (78.1)*	*2 (50.0)*	*4 (100.0)*	3 (42.9)	0.676
Antipyretics; n (%)	33 (70.2)	28 (70.0)	*22 (68.8)*	*2 (50.0)*	*4 (100.0)*	5 (71.4)	0.452
Catecholamines; n (%)	13 (27.7)	13 (32.5)	*11 (34.4)*	*1 (25.0)*	*1 (25.0)*	0 (0.0)	1.000
Inhalation therapy; n (%)	13 (27.7)	13 (32.5)	*11 (34.4)*	*2 (50.0)*	*0 (0.0)*	0 (0.0)	1.000
Oxygen; n (%)	31 (66.0)	28 (70.0)	*20 (62.5)*	*4 (100.0)*	*4 (100.0.)*	3 (42.9)	0.275
Intratracheal ventilation; n (%)	15 (31.9)	14 (35.0)	*12 (37.5)*	*1 (25.0)*	*1 (25.0)*	1 (14.3)	1.000
CPAP; n (%)	11 (23.4)	9 (22.5)	*7 (21.9)*	*0 (0.0)*	*2 (50.0)*	2 (28.6)	0.084
Other; n (%)	14 (29.8)	13 (32.5)	*10 (31.3)*	*2 (50.0)*	*1 (25.0)*	1 (14.3)	0.247
Outcome							
Sequelae; n (%)	5 (10.6)	4 (10.0)	*3 (9.4)*	*1 (25.0)*	*0 (0.0)*	1 (14.3)	0.548
Death; n (%)	5 (10.6)	5 (12.5)	*5 (15.6)*	*0 (0.0)*	*0 (0.0)*	0 (0.0)	1.000

*n.a.* not available, *vs* versus, *i.v.* intravenous

*Fisher’s Exact test or Mann–Whitney *U*-test, respectively

^a^Clinical data was not available for one influenza A(H1N1)pdm09 and three influenza B patients

^b^Oseltamivir (*n* = 8), oseltamivir plus acyclovir (*n* = 1), acyclovir (*n* = 3), gancyclovir plus valgancyclovir (*n* = 1)

^c^Comparison restricted to influenza A(H1N1)pdm09 patients from Season 1 (S1; Oct10–Sep 11; *n* = 18) and Season 3 (S3; Oct12–Sep 13; *n* = 13)

#### Fatalities and sequelae

Five children (10.6 %), infected with A(H1N1)pdm09, died at an age of 4 to 11 years; four were male patients (Table 6). Four of these children suffered both from severe neurological conditions (two children with previous peripartal asphyxia and spastic tetraparesis; one child with cerebral paresis and tetraspasticity; one child with congenital cerebral disorder), and from chronic pulmonary conditions; two of these four children were also born pre-term. Influenza-associated pneumonia was diagnosed in all four of these children; three additionally had secondary bacterial pneumonia, and one child also developed sepsis. For the fifth child, obesity was reported as the only risk factor; and sepsis and suspected encephalitis as complications. Bacterial co-pathogens were detected in three of these five children and suspected in one child; two viral and two fungal co-infections were also reported. All five children received intratracheal ventilation, antibiotics and catecholamines; two were additionally treated with antiviral medication. Death occurred 1, 2, 4, 19, and 26 days after PICU admission, with ARDS reported as cause of death in three children.

**Table 6:** Fatalities in five out of 47 patients with PCR-confirmed influenza in Bavarian PICUs, 2010–2013

Patient	PICU admittance (season)	Age (years)	Influenza type/subtype	Co-pathogen(s) detected^a^	Underlying chronic condition	Influenza vaccination	Complications	Antiviral treatment (days)	Antibiotics i.v. (days)	Cause of death
1	2010/11	10	A(H1N1)pdm09	*Escherichia coli*	Obesity	No	ARDS, sepsis, convulsion (encephalitis suspected)	Acyclovir (2)	Yes (2)	ARDS, Sepsis
2	2010/11	4	A(H1N1)pdm09	*Streptococcus* sp., *Candida* sp.	Neurologic	No	Pneumonia, secondary bacterial pneumonia, pleural effusion	No	Yes (10)	Circulatory insufficiency
					Pulmonal					
					Pre-term birth					
3	2010/11	4	A(H1N1)pdm09	RSV *Pseudomomas aeruginosa*	Neurologic	No	Pneumonia, secondary bacterial pneumonia, ARDS	No	Yes (19)	ARDS
					Pulmonal					
					Pre-term birth					
					Obesity					
4	2012/13	11	A(H1N1)pdm09	RSV, *Candida *sp.	Neurologic	No	Pneumonia, secondary bacterial pneumonia, bronchitis, ARDS, sepsis	Oseltamivir (5)	Yes (22)	ARDS, circulatory insufficiency
					Pulmonal					
5	2012/13	11	A(H1N1)pdm09	None	Neurologic	No	Pneumonia	No	Yes (4)	Respiratory failure
					Pulmonal					

^a^Viral co-infections were detected either by local PCR or central multiplex PCR; bacteria and fungi were identified from blood culture or tracheal aspirates (intratracheal ventilation)

Sequelae were reported for five patients (10.6 %): state after surgery due to pleural effusion/empyema in two children; increased oxygen requirements in two children who had previously already received oxygen therapy at home; damage of the lung after high-pressure ventilation in one child.

### Comparison of PICU patients infected with influenza A(H1N1)pdm09 in the first and the third post-pandemic season

**Fig. 1:**
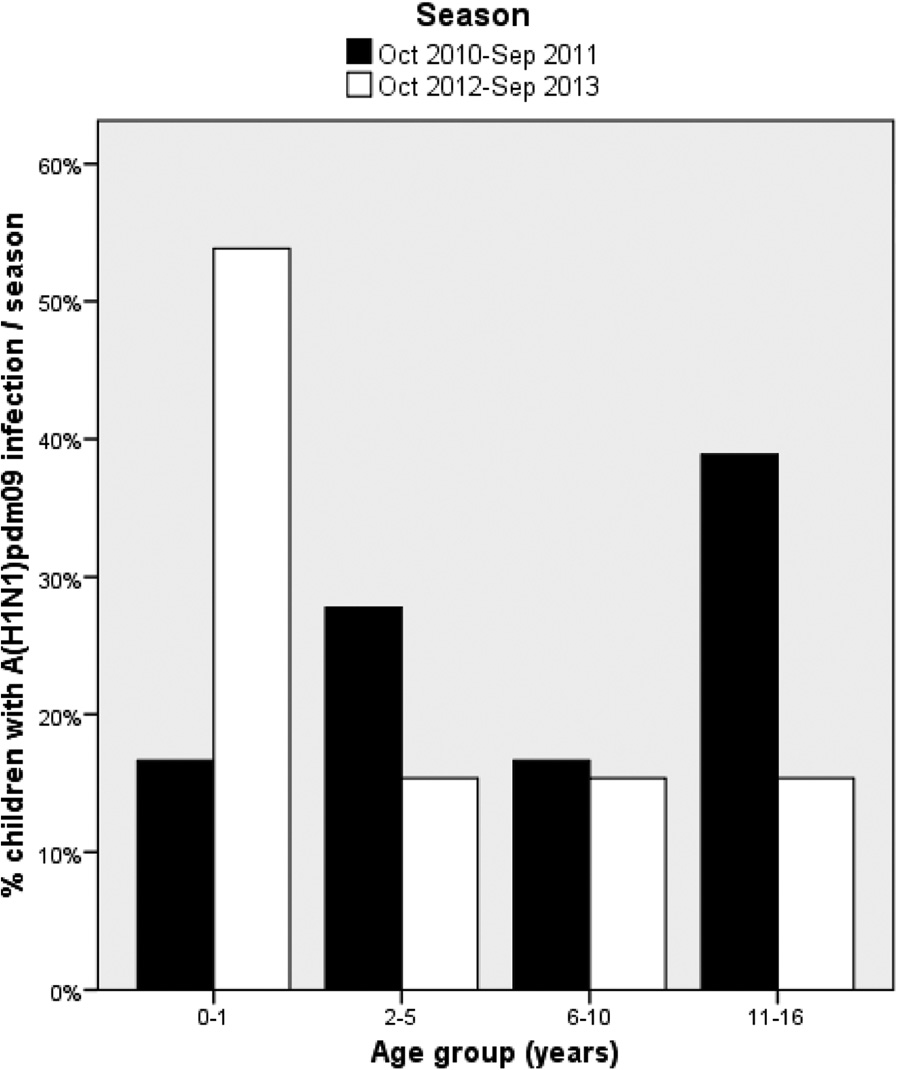
Age distribution of children with PCR-confirmed influenza A(H1N1)pdm09 infection treated in PICUs in Bavaria (figure corresponding to [20]). Data are given in %, by age group and season. Season 1: Oct10–Sep11 (*n* = 18), Season 3: Oct12–Sep13 (*n* = 13); Season 2: Oct11–Sep12 (*n* = 1) is not shown

Influenza A(H1N1)pdm09 from season 1 (*n* = 18) were compared to patients from season 3 (*n* = 13) (Tables 2, 3, 4, 5). Patients with influenza A(H1N1)pdm09 were significantly older in season 1 (median 7.1, IQR 3.3.–12.5) than in season 3 (median 1.7; IQR 0.1–8.3; Table 2). Figure 1 shows the difference in age distribution between both seasons, and the high proportion of children below 2 years of age as opposed to low proportions in all other age groups in season 3. After onset of symptoms, children were admitted to a PICU after a significantly shorter period, with a median of 3 days (IQR 1–4) in season 1 compared to 6 days (IQR 2.0–7.5) in season 3 (Table 2). In season 1, significantly fewer children were diagnosed with bronchitis/bronchiolitis (Table 4), and they tended to require CPAP treatment less frequently than in season 3 (11.1 % vs. 41.7 %, *p* = 0.084, Table 5).

### Comparison of PICU patients infected with influenza in pre-pandemic and post-pandemic seasons

In the pre-pandemic period, median duration of PICU stay was longer (19 days), and children were more often diagnosed with encephalitis/encephalopathy (25 %) and co-infections (65 %) than in later periods (Table 7). The proportion of children with influenza-associated pneumonia was highest (74 %) during the pandemic, whereas secondary bacterial pneumonia (17 %), bronchitis/bronchiolitis (12 %) and sepsis (6 %) were reported less frequently during the pandemic than in the pre- and post-pandemic seasons. Oseltamivir treatment decreased significantly in the post-pandemic period (Table 7).

**Table 7:** Summary of key variables from studies of PICU-associated influenza in Germany, 2005–2013. Data were collected in Germany during the pre-pandemic period 2005/06–2007/08 [18], in Germany during the pandemic season 2009/10 [19], and in Bavaria (Southern Germany) during the post-pandemic period 2010/11–2012/13 (this study)

Key variables of PICU patients	Pre-pandemic (2005/06–2007/08)	Pandemic (2009/10)	Post-pandemic (2010/11–2012/13)	Pre-pandemic vs. post-pandemic^b^
	*N* = 20	*N* = 89^a^	*N* = 47	*p*-value
Male sex; n (%)	12 (60)	56 (60)	28 (60)	1.000
Age at ICU admission, in years (median, IQR)	7.5 (0.5–12.75)	4.5 (1.3–9.3)	4.8 (1.6–11.0)	0.727
Duration of ICU stay, in days (median, IQR)	19 (12–38)	8 (3–17)	3 (1–11)	0.006
Chronic underlying conditions; n (%)	11 (55)	63 (74)	36 (77)	0.089
Complications/diagnoses				
Influenza-associated pneumonia; n (%)	12 (60)	66 (74)	29 (62)	1.000
Bronchitis/bronchiolitis; n (%)	6 (30)	11 (12)	15 (32)	1.000
Secondary bacterial pneumonia; n (%)	5 (25)	15 (17)	12 (26)	1.000
ARDS; n (%)	5 (25)	22 (25)	10 (21)	0.756
Sepsis; n (%)	3 (15)	5 (6)	6 (13)	1.000
Encephalitis/encephalopathy; n (%)	5 (25)	6 (7)	1 (2)	0.008
Co-infections; n (%)	13 (65)	n.a.	16 (34)	0.030
Oseltamivir treatment; n (%)	10 (50)	51 (61)	9 (19)	0.017
Mechanical ventilation; n (%)	10 (50)	56 (65)	22 (47)	1.000
Death; n (%)	2 (10)	11 (12)	5 (11)	1.000
Influenza A(H1N1)pdm09; n (%)	0 (0)	89 (100)	32 (68)	<0.001

^a^89 cases with PICU treatment; proportions may refer to slightly lower patient numbers, see Altmann et al. 2011 [19]

^b^Key data from this study were pooled with data from Streng et al. 2011 [18] and compared using Fisher’s Exact test or Mann–Whitney *U*-test, respectively

## Discussion

During the first three post-pandemic seasons 2010/11, 2011/12 and 2012/13, active screening of children with acute respiratory infection admitted to 24 paediatric intensive care units in Bavaria identified a total of 51 PCR-confirmed influenza cases, resulting in annual incidence estimates of 1.7, 0.7, and 1.8 influenza-associated PICU admissions per 100,000 children, respectively. These figures would, by extrapolation, correspond to a total number of 559 children with influenza-associated PICU admission in Germany within the 3-year post-pandemic period, with an annual average of 186 children. This is almost 28 times as high as the annual average of six to seven influenza-associated PICU admissions detected by nation-wide PICU surveillance in Germany during three pre-pandemic years without active screening [18]. Furthermore, the incidence estimates for the subtype A(H1N1)pdm09 derived from our active screening study were higher in the first and third post-pandemic seasons (1.35 and 1.17, respectively) than previous incidence estimates for PICU patients in the pandemic (0.8–1.0) and the first post-pandemic (approximately 0.4) season in Germany [19, 20]. Thus, our results indicate possible underreporting in previous studies, and show a continued high level of A(H1N1)pdm09-associated PICU admissions even 3 years after the pandemic.

In our study, the proportions of children with bacteria-associated complications (secondary bacterial pneumonia, sepsis) were similar to the proportions observed during the pre-pandemic period, but appeared higher than those observed during the pandemic 2009/10 [19]. The lower proportions observed during the pandemic might be explained by the time shift of the peak of influenza cases, which was observed as early as November 2009 in Germany [19]. Thus, the pandemic influenza peak did not coincide with the seasonal peak of *Streptococcus pneumoniae*, the bacterial pathogen most frequently associated with community-acquired influenza [23].

Antiviral treatment patterns changed considerably during the post-pandemic period, with a decrease in the proportion of paediatric influenza cases receiving oseltamivir from previously 50 % [18] and 61 % [19] to 19 %. Oseltamivir is considered to be most advantageous when administered within the first 48 h of influenza disease. The reduced use in the post-pandemic period may therefore be partly due to the fact that median time between onset of influenza symptoms and PICU admission was longer than during the pandemic (3 vs. 2 days [19]). Increasing uncertainty regarding the effectiveness of oseltamivir in the treatment of paediatric influenza may also have played a role [24, 25]. Post-pandemic oseltamivir treatment was associated with co-infections and longer PICU stay, suggesting that it were mainly children with severe complications or with serious underlying conditions who received this medication.

In our study, about two-thirds of influenza cases and all fatalities were A(H1N1)pdm09-associated. During the post-pandemic seasons 1/2/3, the proportion of A(H1N1)pdm09 cases among the PICU patients was 79 %/14 %/65 % and, thus, considerably higher than the proportion of this subtype reported among outpatients by national influenza surveillance (65 %/1 %/34 %) [17]. This observation suggests that A(H1N1)pdm09 may be associated more often with a severe course of influenza requiring PICU treatment than other influenza types/subtypes. Similar observations on the proportion of A(H1N1)pdm09-associated PICU admissions have been reported in the United States [9].

Comparison of PICU patients with A(H1N1)pdm09 between the post-pandemic seasons showed that median age was 1.7 years in the third season and, thus, significantly lower than in the first season. A significant age shift towards younger children, from a median age of 5 to 3 years, had already been observed in a comparison of the pandemic and the first post-pandemic season in Germany [20]. The continued shift towards younger patients in the third season is likely to be due to increasing immunity in the older paediatric population, after previous contact with A(H1N1)pdm09. Seroprevalence data from Germany had already shown evidence for A(H1N1)pdm09 infection in as many as 25 % of children aged 1–4 years and 48 % of 5–17 year-old children for the pandemic season 2009/10 [26]. A similar shift towards younger hospitalized children [27, 28] and towards younger children with severe paediatric A(H1N1)pdm09-associated influenza from the pandemic season to the first post-pandemic season had also been detected in other European countries [28–31].

In Germany, paediatric influenza vaccination for pandemic influenza A(H1N1)pdm09 was recommended and funded for all children as monovalent vaccination from October 2009 to July 2010 [32]. For seasonal influenza, however, paediatric influenza vaccination was and is currently recommended only for specific risk groups with underlying chronic conditions [33]. Vaccination uptake was low, even in this target group. Pre-pandemic vaccination rates were 5 % for all children and about 15 % for children with chronic underlying conditions in 2007/2008 [34]. For the pandemic and post-pandemic seasons, no data on vaccination rates is available for children, but vaccination rates as low as 14 % (2009/10) and 11 % (2010/11) were reported for adults, with a vaccination rate of only 17 % even for risk group adults [35]. In our study, more than 75 % of influenza-associated PICU patients were children with underlying chronic conditions. Analysis of their reported influenza vaccination status showed that among these were a high proportion of vaccine-eligible but unvaccinated children. Patients with chronic conditions too young to be vaccinated and other paediatric risk groups, such as otherwise healthy children below 2 years of age, are not covered by the current German recommendation. All these groups could profit considerably from universal influenza vaccination for children, either directly or by herd protection. In contrast to the situation in Germany, in the United States universal influenza vaccination for all children older than 6 months of age has been established, and vaccination coverage reached a level of approximately 41 % in 2013 [36]. Compared to 348 influenza-associated paediatric deaths observed in the United States during the pandemic 2009/10, only 79 were observed in the strong A(H1N1)pdm09 season 2013/14 [37]. This might partly be explained by increasing immunity in children after previous A(H1N1)pdm09 infection, but may in part also be a result of the influenza vaccination program [37]. In England, a universal childhood vaccination programme with a new live attenuated influenza vaccine (LAIV) with intra-nasal application was started in the 2013/14 influenza season [38]. First results showed an overall uptake of 53 % in primary school aged children, indicating a good acceptance of LAIV, and suggesting direct and indirect impacts on disease incidence, including reduction of paediatric influenza-associated hospitalisations.

To our knowledge, our study is the first in Europe to investigate paediatric influenza in PICU patients during the first three post-pandemic seasons after the 2009/10 pandemic. The strengths of our study include the multi-centre design covering the majority of PICUs in Bavaria, the active screening for influenza in patients admitted to PICUs, and PCR-confirmation of all influenza cases. A limitation is that the corrected incidence estimates were based on the assumption that participating and non-participating PICUs treated a similar number of severe paediatric influenza patients. Although PICUs of both groups were of similar size, some of the non-participating PICUs, where paediatricians indicated lack of time as reason for non-participation, may have treated a higher number of patients, or patients with higher acuity. Further limitations include potential over- and underreporting in participating PICUs. On the one hand, due to different hospitalization rules some children may have been admitted to PICUs mainly for the purpose of monitoring their course of influenza disease more closely, thus resulting in an overestimate of severe cases. On the other hand, some parents of children with severe influenza may have refused study participation, or children with a fulminant course of influenza disease may have died before they were admitted to a PICU [4]. Thus, the high incidence estimates derived in this study may still underestimate the true burden of severe influenza.

## Conclusions

The incidence estimates of influenza A(H1N1)pdm09-associated PICU admissions, derived from active screening of PICU patients with acute respiratory infections, reached similarly high levels in the first and third post-pandemic seasons. Both incidence estimates were higher than those previously reported by nation-wide PICU surveillance for the pandemic and the first post-pandemic season, suggesting possible underreporting in previous studies without active screening. Comparison of the first and third post-pandemic seasons indicated an age shift of severe A(H1N1)pdm09 towards younger children, which might be explained by increasing immunity in the older paediatric population. The large proportion of children with underlying chronic conditions indicates the need for a more consistent implementation of the current recommendations for influenza vaccination of specific risk groups in Germany. These children could also profit from herd protection, if universal influenza vaccination was successfully introduced in Germany.

## Abbreviations

AdV: adenovirus
ARDS: acute respiratory distress syndrome
ARI: acute respiratory infection
CoV: coronavirus
CPAP: continuous positive airway pressure
ECMO: extracorporeal membrane oxygenation
EV: enterovirus
hBoV: human bocavirus
hMPV: human metapneumovirus
ICU: intensive care unit
IQR: inter-quartile range
i.v.: intravenous
PCR: polymerase chain reaction
PICU: paediatric intensive care unit
PIV: parainfluenza virus
PV: parechovirus
RhV: rhinovirus
RSV: respiratory syncytial virus
sp.: species
